# The role of Executive Attention in the association between obsessive-compulsive symptoms and relapses in Major Depressive and Bipolar Disorder

**DOI:** 10.1192/j.eurpsy.2022.423

**Published:** 2022-09-01

**Authors:** L. Lucassen, I. Tioli, M. Ferrari, P. Ossola, C. Marchesi

**Affiliations:** 1 University of Parma, Department Of Medicine And Surgery, Parma, Italy; 2 AUSL of Parma, Department Of Mental Healthy, Parma, Italy; 3 University of Parma, Department Of Medicine & Surgery, Unit Of Neuroscience, Psychiatric Unit, University Of Parma, Parma, Italy, Parma, Italy

**Keywords:** bipolar disorder, Obsessive-compulsive symptoms, Executive Attention, major depressive disorder

## Abstract

**Introduction:**

Major Depressive (MDD) and Bipolar Disorder (BD) are chronic relapsing condition in which mood episodes are interspersed with periods of euthymia. Impairments in Executive Attention (EA) are a trait characteristic of mood disorder that persists also during remission. Similarly prefrontal dysfunctions are crucial in the genesis and maintenance of Obsessive-Compulsive Symptoms (OCS), which are highly comorbid in both MDD and BD.

**Objectives:**

The aim of this study is to test a model in which deficits in EA mediate the relationship between the OCS and the relapse in a cohort of patients with MDD and BD.

**Methods:**

Sixty-four euthymic subjects with BD and MDD performed the Attentional Network Task Revised (ANT-R), that gauges EA in a standard conflict task. Here we adopted a drift diffusion model to measure the task efficiency as the drift rate in incongruent trials. Patients also completed at baseline the YBOCS, a questionnaire that evaluate the severity of OCS. All the participants have been followed-up for up to 5 years and relapses have been recorded.

**Results:**

The association between OCS and time in euthymia was fully mediated by the EA so that greater OCS were associated with poorer executive functions (beta=-0.341; p=0.006) that in turn predicted a sooner relapse (beta=0.349; p=0.005). This held true even when controlling for classic predictors of recurrence such as previous episode distance, the duration of illness and medications.

**Conclusions:**

Treatment targeting executive functions could hence be crucial in preventing relapses in subjects with mood disorders experiencing obsessive compulsive-symptoms.

**Disclosure:**

No significant relationships.

